# Supplementary material for: A Comprehensive Assessment of Ultraviolet-Radiation-Induced Mutations in Flammulina filiformis Using Whole-Genome Resequencing
Source: J Fungi (Basel). 2024 Mar 20;10(3):228. doi: 10.3390/jof10030228 (PMC10971301; doi:10.3390/jof10030228)
Supplement: Supplementary file 1 [file jof-10-00228-s001.zip › Supplementary Material S8/KEGG annotation/out/64381550635650.os/KO/out_map/map04540.html]

KEGG PATHWAY: Gap junction - Reference pathway


|  |  |
| --- | --- |
| **Gap junction - Reference pathway** |  |

[
Pathway menu
| Organism menu
| Pathway entry
| Show description
| User data mapping
]

Gap junctions contain intercellular channels that allow direct communication between the cytosolic compartments of adjacent cells. Each gap junction channel is formed by docking of two 'hemichannels', each containing six connexins, contributed by each neighboring cell. These channels permit the direct transfer of small molecules including ions, amino acids, nucleotides, second messengers and other metabolites between adjacent cells. Gap junctional communication is essential for many physiological events, including embryonic development, electrical coupling, metabolic transport, apoptosis, and tissue homeostasis. Communication through Gap Junction is sensitive to a variety of stimuli, including changes in the level of intracellular Ca2+, pH, transjunctional applied voltage and phosphorylation/dephosphorylation processes. This figure represents the possible activation routes of different protein kinases involved in Cx43 and Cx36 phosphorylation.

|  |  |
| --- | --- |
| Reference pathway | 100% |
